# Dosimetry of oblique tangential photon beams calculated by superposition/convolution algorithms: a Monte Carlo evaluation

**DOI:** 10.1120/jacmp.v12i1.3424

**Published:** 2010-11-03

**Authors:** James C. L. Chow, Runqing Jiang, Michael K. K. Leung

**Affiliations:** ^1^ Department of Radiation Oncology Princess Margaret Hospital, University Health Network Toronto ON Canada; ^2^ University of Toronto and Radiation Medicine Program, Princess Margaret Hospital, University Health Network Toronto ON Canada; ^3^ Department of Physics Ryerson University Toronto ON Canada; ^4^ Medical Physics Department Grand River Regional Cancer Center Kitchener ON Canada; ^5^ Department of Medical Biophysics University of Toronto ON Canada

**Keywords:** anisotropic analytical algorithm, collapsed cone convolution, MC simulation, dosimetry, inhomogeneity correction, surface dose

## Abstract

Although there are many works on evaluating dose calculations of the anisotropic analytical algorithm (AAA) using various homogeneous and heterogeneous phantoms, related work concerning dosimetry due to tangential photon beam is lacking. In this study, dosimetry predicted by the AAA and collapsed cone convolution (CCC) algorithm was evaluated using the tangential photon beam and phantom geometry. The photon beams of 6 and 15 MV with field sizes of 4×4 (or 7×7), 10×10 and $20\times 20\;{\text{cm}}^{2}$, produced by a Varian 21 EX linear accelerator, were used to test performances of the AAA and CCC using Monte Carlo (MC) simulation (EGSnrc‐based code) as a benchmark. Horizontal dose profiles at different depths, phantom skin profiles (i.e., vertical dose profiles at a distance of 2 mm from the phantom lateral surface), gamma dose distributions, and dose‐volume histograms (DVHs) of skin slab were determined. For dose profiles at different depths, the CCC agreed better with doses in the air‐phantom region, while both the AAA and CCC agreed well with doses in the penumbra region, when compared to the MC. Gamma evaluations between the AAA/CCC and MC showed that deviations of 2D dose distribution occurred in both beam edges in the phantom and air‐phantom interface. Moreover, the gamma dose deviation is less significant in the air‐phantom interface than the penumbra. DVHs of skin slab showed that both the AAA and CCC underestimated the width of the dose drop‐off region for both the 6 and 15MV photon beams. When the gantry angle was 0°, it was found that both the AAA and CCC overestimated doses in the phantom skin profiles compared to the MC, with various photon beam energies and field sizes. The mean dose differences with doses normalized to the prescription point for the AAA and CCC were respectively:7.6%±2.6% and 2.1%±1.3% for a $10\times 10\;{\text{cm}}^{2}$ field, 6 MV; 16.3%±2.1% and 6.7%±2.1% for a $20\times 20\;{\text{cm}}^{2}$ field, 6 MV; 5.5%±1.2% and 1.7%±1.4% for a $10\times 10\;{\text{cm}}^{2}$, 15 MV; 18.0%±1.3% and 8.3%±1.8% for a $20\times 20\;{\text{cm}}^{2}$, 15 MV. However, underestimations of doses in the phantom skin profile were found with small fields of 4×4 and $7\times 7\;{\text{cm}}^{2}$ for the 6 and 15 MV photon beams, respectively, when the gantry was turned 5° anticlockwise. As surface dose with tangential photon beam geometry is important in some radiation treatment sites such as breast, chest wall and sarcoma, it is found that neither of the treatment planning system algorithms can predict the dose well at depths shallower than 2 mm. The dosimetry data and beam and phantom geometry in this study provide a better knowledge of a dose calculation algorithm in tangential‐like irradiation.

PACS numbers: 87.55.‐x, 87.53.Bn, 87.55.K‐, 87.55.kh, 87.56.jf

## I. INTRODUCTION

Modern dose escalation techniques such as image‐guided intensity‐modulated radiation therapy (IMRT) requires a precise, accurate and fast dose calculation algorithm that can generate reliable dose distributions and dose‐volume information for treatment planning and evaluation.^(^ ^1^ ^–^ ^4^ ^)^ Semi‐analytical algorithms for dose calculation such as the superposition/convolution method^(^ ^5^ ^–^ ^7^ ^)^ are currently used by most of commercial treatment planning systems (TPSs), and have proved to be efficient and accurate in the routine treatment planning practice.^(^ ^8^ ^–^ ^12^ ^)^

In all superposition/convolution methods, the collapsed cone convolution (CCC) algorithm^(^ ^13^ ^)^ and anisotropic analytical algorithm^(^ ^14^ ^,^ ^15^ ^)^ (AAA) are popular, and have a good accuracy in heterogeneous media. There were a number of evaluations concerning dose calculations of the AAA and CCC in the heterogeneous media using various heterogeneous and homogeneous phantoms.^(^ ^16^ ^–^ ^25^ ^)^ However, the above studies concern mostly the lung and bone doses using different radiation treatment techniques such as IMRT, stereotactic body radiation therapy, and intensity‐modulated arc therapy. Among these studies, only Panettieri et al.^(^ ^21^ ^)^ studied the AAA and pencil‐beam convolution (PBC) calculation accuracy in the buildup region using a cylindrical phantom with the 6 and 18 MV photon beams. The authors reported that both algorithms yield equivalent results after the first 2 mm of tissue from the surface using the 6 MV photon beams. It can therefore be seen that related phantom study concerning the evaluation of surface dosimetry for the AAA and CCC is desired.

Performances of the AAA and CCC in calculating surface doses were evaluated using the MC simulation as a benchmark for comparison. A tangential photon beam, with its central beam axis (CAX) parallel to the solid water phantom lateral surface (skin) was used. Moreover, the photon beam was rotated anticlockwise around the isocenter. In this study, horizontal dose profiles at different depths (1D), gamma dose distribution comparisons between the AAA/CCC and MC (2D), and dose‐volume histograms (DVHs) of skin slab on the phantom lateral surface (3D) were carried out. In addition, doses along the CAX at 2 mm distance from the phantom lateral surface (phantom skin profile) were calculated using the AAA, CCC and MC. The aim of this study is to evaluate the dose calculation performances of the AAA and CCC under different tangential beam geometries so as to have a better knowledge of the dose calculation algorithm for our routine treatment planning practice.^(^ ^26^ ^)^

## II. MATERIALS AND METHODS

### A. Phantom and beam geometry

**Figure 1:**
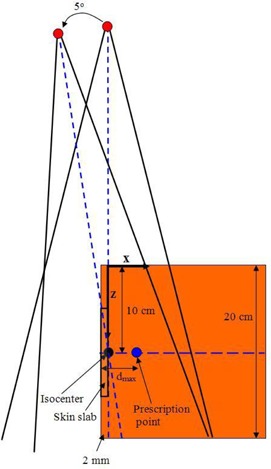
Schematic diagram (not to scale) showing the calculated configuration of the solid water phantom. The photon beams of 6 and 15 MV with field sizes of 4×4 (6 MV), 7×7 (15 MV), 10×10 and $20\times 20\;{\text{cm}}^{2}$ are used with SAD=100 cm. Photon beams are rotated 5° and 45° anticlockwise around the isocenter at a depth of 10 cm. $d_{\text{max}}$ is the depth of maximum dose. Both photon beams with gantry angles 0° and 5° are shown in the figure.

The calculated setup for the phantom and photon beam is shown in Fig. 1. The photon beams of 6 and 15 MV with field sizes of 4×4, 7×7, 10×10, and $20\times 20\;{\text{cm}}^{2}$ produced by a Varian 21EX linear accelerator (linac) (Varian Medical Systems, Palo Alto, CA) were used. The CAX of the photon beam (short‐broken line) was parallel to the phantom lateral surface, as shown in Fig. 1. The isocenter was at a depth of 10 cm and a distance of 2 mm from the right phantom lateral surface. The dimension of the phantom was $20\times 20\times 20\;{\text{cm}}^{3}$ with the source‐to‐axis distance (SAD) equal to 100 cm. The positive x‐, y‐ and z‐axes are pointed toward the right‐hand side of the phantom, out of Fig. 1 and the bottom of the phantom, respectively. Doses along the vertical and horizontal broken lines (Fig. 1) were calculated using the AAA, CCC and MC. The dose prescription or normalization point was set at the depths of maximum dose (i.e., $d_{\max}=1.5\;\text{cm}$ for 6 MV and 3 cm for 15 MV) along the horizontal axis in the phantom, as shown in Fig. 1. It should be noted that a larger field of $7\times 7\;{\text{cm}}^{2}$ (instead of $4\times 4\;{\text{cm}}^{2}$) was used for the 15 MV photon beams to ensure that the normalization point was within the field at a depth of 10 cm, when the gantry angle is equal to 0°. The reason for the 2 mm distance of doses along the CAX is that such distance is typically within the human skin layer range, because the thickness of dermis is about 0.5–2 mm. In this study, doses along the CAX (vertical broken line in Fig. 1) are referred to the phantom skin profiles. Apart from positioning photon beams at gantry angle equal to 0°, beams were rotated 5° and 45° anticlockwise, and then the same dose calculations and MC simulations were repeated. It should be noted that when the gantry is rotated 5° or more with an isocentric geometry having the isocenter at 10 cm depth, the CAX enters the phantom from the lateral surface.

### B. Dose calculations using the AAA and CCC

#### B.1 The AAA and CCC algorithm

The AAA is a 3D pencil beam convolution/superposition algorithm.^(^ ^14^ ^,^ ^15^ ^)^ The pencil beam used MC simulations with adjustment based on measurement to consider the primary photons, scattered extrafocal photons and scattered electrons. The longitudinal distribution of the pencil beam is scaled using the equivalent path length method, and the lateral distribution of the pencil beam is scaled according to the equivalent path length to the calculation point based on the densities relative to water in the previous layer of the irradiated volume. Therefore, the AAA accounts for the tissue heterogeneity using lateral scaling in a plane normal to the propagation of the pencil beam. The lateral plane has a spherical shape with the center located in the beam target. In the AAA, electron contamination is modeled with a depth‐dependent curve that describes the total amount of electron contamination dose at a certain depth. Moreover, electron contamination is used to model the photon contamination.^(^ ^27^ ^)^

The CCC algorithm determines the total energy released per unit mass (TERMA) in a 3D matrix of the irradiated volume, based on the ray‐tracing technique.^(^ ^13^ ^)^ The TERMA contained two parts namely, the primary (collision kerma) and scatter (difference between TERMA and collision kerma) part. Dose in voxel of the irradiated volume is then determined by convolving the point kernel with the TERMA distribution. Heterogeneities are considered by scaling the point kernel model in 106 different directions per the elemental composition and density variations in the medium. Contaminant electrons entering to the phantom in the buildup region, is modeled by ${\text{Pinnacle}}^{3}$ using an exponential function. This contaminated electron dose is added to the photon dose.^(^ ^28^ ^)^

Unlike the MC simulation, analytical dose calculations using the AAA and CCC algorithm use only an approximate model (e.g., direct particle transport in the phantom) to describe the electron transport. Phenomena in radiation transport such as charge particle equilibrium and photon scatter equilibrium could therefore be a concern in our tangential beam geometry.

#### B.2 Dose calculation in TPS

The AAA and CCC were available in the Eclipse (version 8.5, Varian Medical Systems, Palo Alto, CA) and ${\text{Pinnacle}}^{3}$ (version 7.4, Philips Medical Systems, Andover, MA) TPS, respectively. Both dose calculation algorithms were configured and verified using the same set of commissioning data generated by a scanning water tank (RFA 300, Scanditronix Medical AB, Bartlett, TN) at the Grand River Regional Cancer Center. The water tank was controlled by the OmniPro 6 software so that the beam profiles (i.e., dose profiles perpendicular to the CAX) and depth doses could be measured by a servo motor system with a photon diode detector (PDF‐3G, Scanditronix Medical AB). The photon beam output data for commissioning were measured by a micro‐ionization chamber (RK8304, Scanditronix Medical AB) having an air cavity volume of $0.12\;{\text{cm}}^{3}$. After the TPS/algorithm commissioning phase based on the manufacturers' commissioning guides,^(^ ^27^ ^,^ ^28^ ^)^ the dose calculation accuracy should reach the acceptability criterion of 1%/2 mm for both the AAA and CCC method.

To calculate doses along the vertical and horizontal axes (broken lines) as shown in Fig. 1, phantom and beam geometries were input into the two TPSs with the dose grid set at 1 mm. Doses in the phantom for the photon beams (6 and 15 MV; 4×4 (or 7×7), 10×10 and $20\times 20\;{\text{cm}}^{2}$) with gantry rotated 5° and 45° anticlockwise were calculated using the AAA and CCC.

### C. MC simulation

The EGSnrc‐based code (version 4‐r2‐2‐5), developed by the National Research Council Canada, was used in this study.^(^ ^29^ ^,^ ^30^ ^)^ The code was run on a personal computer with a single INTEL Core2 Quad processor with 2.4 GHz using 3 GB of RAM. BEAMnrc and DOSXYZnrc associated with EGSnrc were used to generate phase space beams and calculate doses.^(^ ^31^ ^–^ ^33^ ^)^

#### C.1 Phase space beam of linac

Photon beams of 6 and 15 MV with field sizes of 4×4 (6 MV), 7×7 (15 MV), 10×10 and $20\times 20\;{\text{cm}}^{2}$ produced by a Varian 21 EX linac were modeled to generate phase space files using the BEAMnrc code. Details of geometries and materials of different components in the linac head were provided by the manufacturer. For the electron source with energy distribution as a Gaussian with a FWHM of 1 MeV, the distribution was centered at 6 and 15 MeV for the 6 and 15 MV photon beams, respectively. The FWHM focal spot size was 1.5 mm for the 6 and 15 MV photon beams. Statistical uncertainties on MC simulations are at the one standard deviation level.^(^ ^34^ ^)^

Each of six phase space files contained $4\times 10^{7}$ particles. For transport parameters, the electron and photon cutoff energy were set at 700 and 10 keV, respectively. Parameter reduced electron step transport algorithm (PRESTA) II was used as the electron‐step algorithm, and all user‐adjustable parameters in PRESTA were set at their default values.^(^ ^30^ ^,^ ^35^ ^)^

**Figure 2:**
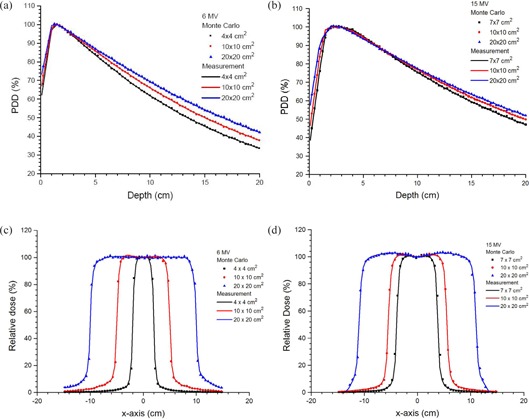
Percentage depth dose curves for: 6 (a) and 15 MV (b) photon beams; beam profiles at 10 cm depth for 6 (c) and 15 MV (d) photon beams. Field sizes of 4×4 (6 MV), 7×7 (15 MV), 10×10 and $20\times 20\;{\text{cm}}^{2}$ were measured using the photon diode detector and calculated using MC simulations in water.

Verifications of phase space files or beams were done by comparing the percentage depth doses (PDDs) and beam profiles calculated by MC simulations (with DOSXYZnrc) to measurements from the linac commissioning. Figures 2(a)–2(d) show the PDD curves and beam profiles at a depth of 10 cm with source‐to‐surface distance (SSD) equal to 100 cm for the 6 and 15MV photon beams with different field sizes, calculated using the MC simulation, and measured using the scanning water tank with photon diode detector. The measurements in Figs. 2(a)–2(d) are based on the commissioning data of the ${\text{Pinnacle}}^{3}$ and Eclipse. It can be seen that deviations <±1% were found between results of MC simulations and measurements for the PDDs as shown in Figs. 2(a) and 2(b) for the 6 and 15 MV photon beams, respectively. In this study, the commissioning results were used for the MC verification only. It should be noted that the TPS commissioning was only based on the dosimetry measurement but MC simulation.

For beam profiles in Figs. 2(c) and 2(d) for the 6 and 15 MV photon beams, the deviations between the measurement and MC simulations are within ±2%, except in the penumbra region where the dose gradient is large. More MC verification results concerning the tangential photon beams can be found in our previous work.^(^ ^36^ ^)^ Therefore, phase space beams were verified in this study.

#### C.2 Dose calculation using DOSXYZnrc

Phantom and beam geometries as shown in Fig. 1 were input to the DOSXYZnrc for dose calculations using the 6 and 15 MV phase space beams with different field sizes. The voxel size of the phantom was set at $0.133\;\times 0.4\times 0.4\;{\text{cm}}^{3}$ corresponding to the X, Y and Z directions, as shown in Fig. 1. The direction of the positive y‐axis in Fig. 1 was pointing out of the figure. Using this voxel size configuration, the phantom skin profile along the CAX (Fig. 1) was exactly in the middle of the second voxel column (i.e., 2 mm) from the right lateral surface of the phantom. This calculation depth (2 mm) of the phantom skin profile is therefore equal to the second layer of the dose calculation grids for the AAA and CCC. Moreover, a relatively larger MC voxel can enhance the sampling rate of ionization. Doses along the vertical and horizontal broken lines in the phantom (Fig. 1) for the 6 and 15 MV photon beams with different field sizes and gantry angles were calculated using MC simulations. The electron and photon cutoff energy were set to 700 and 10 keV, respectively. Five hundred million histories were run in each calculation by recycling particles in the phase space file. For this number of histories, the relative dose error (i.e., the statistical uncertainty as a fraction of the dose in voxel) was within ±1% according to our EGSnrc dose output file.^(^ ^33^ ^)^

### D. Gamma and DVH analysis

Gamma analysis using the software tool developed by Low et al.^(^ ^37^ ^)^ was carried out to determine the gamma quantity, representing the minimum distance in the renormalized multidimensional space between the evaluated distribution and reference point. The 2D dose distributions calculated by the AAA, CCC and MC with the beam setup as in Fig. 1 using the 6 and 15 MV photon beams were used in the gamma method. The field sizes for the 6 and 15 MV photon beams are 4×4 and $7\times 7\;{\text{cm}}^{2}$, respectively. The dose difference and distance‐to‐agreement criteria for the gamma analysis were set to 2% and 3 mm, respectively. In this study, evaluation distributions were from the AAA and CCC with the MC as a reference.

For the 3D dose calculation results from the AAA, CCC and MC based on the calculated configuration in Fig. 1, DVHs were generated with a skin slab on the phantom lateral surface. The length of the slab (z‐axis) was equal to 10 cm (i.e., 5 cm up and down from the isocenter), and the width of the slab (y‐axis) was equal to 5 cm (i.e., 2.5 cm left and right from the CAX). The thickness of the slab was equal to 2 mm. Moreover, field size of $20\times 20\;{\text{cm}}^{2}$ was used for both the 6 and 15 MV photon beams.

## III. RESULTS & DISCUSSION

### A. Uncertainties of voxel and grid size in the MC, AAA and CCC calculations

In this study, different voxel and grid sizes for the MC and AAA/CCC were used. The voxel size of the MC was slightly bigger than that of the AAA/CCC, as explained in Sec. II.C.2. Although this setting provided an exact depth (2 mm) of the skin dose profile for the MC, AAA and CCC comparison, interpolation of the MC results was needed for the gamma and DVH analysis in order to match the same grid size of the AAA and CCC. The uncertainty due to the dose interpolation should be smaller than 0.5 mm in this study.

An additional uncertainty from the AAA and CCC was from setting the calculation volume on the phantom lateral surface. Unlike the MC simulation (EGSnrc code) that the simulation configuration (Fig. 1) is built on voxels, TPSs (${\text{Pinnacle}}^{3}$ and Eclipse) require the user to outline the calculation volume of the grid in the plan with the phantom image. The selection of such volume is controlled by the user manually, and therefore human variation would affect the validity of the results. In the plan, matching the grid line exactly along the air‐phantom interface would lead to about 0.5 mm uncertainty and this should be added to the acceptability criterion (i.e., 1%/2 mm) of the commissioning phase. In the MC simulation, the dose uncertainty issue due to the grid size of the AAA and CCC can be solved by setting the voxel edge exactly at the phantom lateral surface in the simulation code. However, there is still about 1% uncertainty in the validation of MC results with measurements (Fig. 2). Therefore, the error associated with results in this study was estimated to be 2%/3 mm.

### B. Dose profiles at different depths

**Figure 3:**
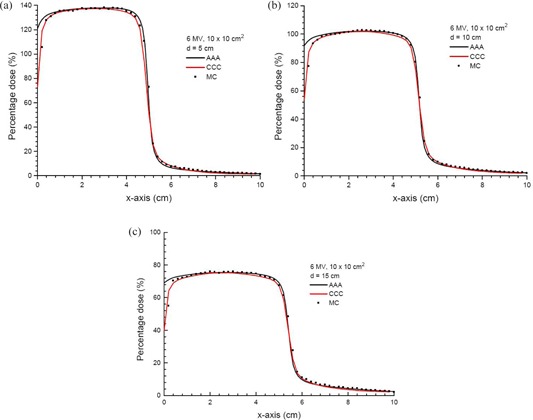
Dose profiles in the x‐axis at depths of 5 (a), 10 (b) and 15 cm (c) in the solid water phantom as shown in Fig. 1. Doses in the profiles are calculated by the AAA, CCC and MC simulations using the 6 MV photon beams ($10\times 10\;{\text{cm}}^{2}$) at gantry angle 0°. All profiles are normalized to the prescription points in Fig. 1.

**Figure 4:**
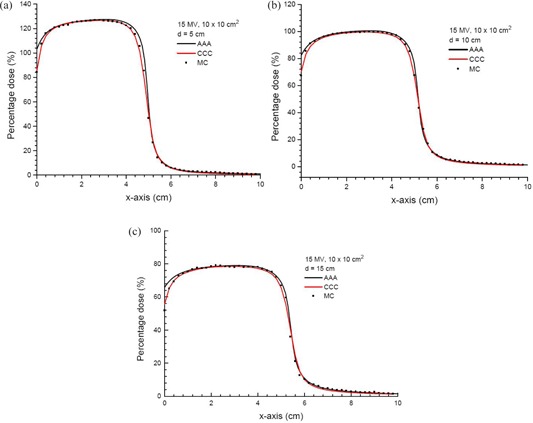
Dose profiles in the x‐axis at depths of 5 (a), 10 (b) and 15 cm (c) in the solid water phantom as shown in Fig. 1. Doses in the profiles are calculated by the AAA, CCC and MC simulations using the 15 MV photon beams ($10\times 10\;{\text{cm}}^{2}$) at gantry angle 0°. All profiles are normalized to the prescription points in Fig. 1.

**Figure 5:**
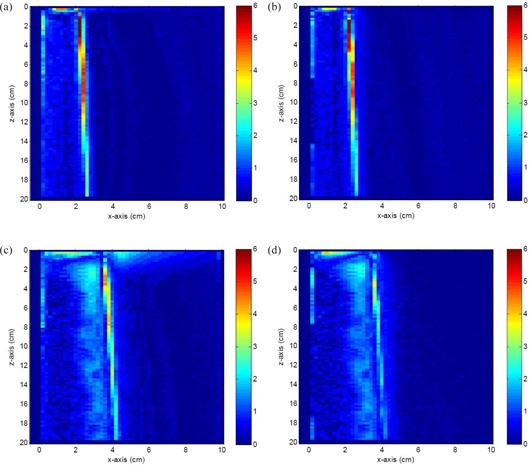
Gamma dose distribution comparisons between the AAA (a) and MC, and the CCC (b) and MC for a 6 MV photon beam ($4\times 4\;{\text{cm}}^{2}$) with gantry angle 0°. Gamma dose distribution comparisons for the 15 MV photon beam ($7\times 7\;{\text{cm}}^{2}$) for the AAA and MC, and the CCC and MC are shown in (c) and (d), respectively.

**Figure 6:**
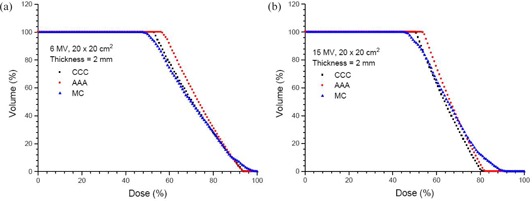
DVHs for skin slab with an area of $10\times 5\;{\text{cm}}^{2}$ along the z‐ and y‐axis, respectively. The thickness of the slab is equal to 2 mm. Photon beams ($20\times 20\;{\text{cm}}^{2}$) with energies of 6 MV (a) and 15 MV (b) were used with gantry angle 0°. Doses were calculated using the AAA, CCC and MC.

Figures 3(a)–3(c) show the dose profiles along the x‐axis at depths of 5, 10 and 15 cm using the 6 MV photon beams ($10\times 10\;{\text{cm}}^{2}$). In Figs. 3–6, all doses calculated by the AAA, CCC and MC were normalized to the prescription points with distances equal to $d_{\text{max}}$ corresponding to the photon beam energies from the phantom lateral surface (Fig. 1). The dosimetry at the normalization point is more stable than dose points in the phantom skin profile, which is just 2 mm from the phantom lateral surface. The horizontal broken line in Fig. 1 represents the dose profiles in Fig. 3(b). The zero coordinate at the x‐axis of Fig. 3 represents the phantom lateral surface (i.e., air‐phantom interface). For the 6 MV photon beams, it can be seen in Fig. 3 that the CCC agrees better than the AAA with MC for doses close to and at the air‐phantom interface. A larger dose drop from the air‐phantom interface is predicted by the CCC and MC, compared to that calculated by the AAA. It can be seen that the CCC can model the transport of energy out from the phantom better than the AAA, which stops the dose integration at the air‐phantom interface, and results in a drop in the dose profile due to the lack of scattered photons at the phantom lateral surface. In the inner penumbra region (i.e., percentage dose between 80% and 50%), it is found that the AAA and CCC slightly overestimate and underestimate doses compared to the MC, respectively. The overestimation of the dose in the inner penumbra region of the AAA is because the algorithm does not handle the dose reduction due to the charge particle equilibrium well in the lateral plane perpendicular to the phantom edge. On the other hand, the CCC slightly underestimates the inner penumbra, which reflects the algorithm's overestimation of the dose reduction from the charge particle equilibrium. The dosimetric difference between the AAA and CCC may be due to their different shapes of lateral part of the pencil beam or point kernals. For the outer penumbra region (i.e., percentage dose between 50% and 20%), the AAA is found to underestimate slightly the dose compared to the MC. However, penumbras at different depths calculated by the AAA and CCC have positional deviations smaller than 2 mm at the same percentage dose compared to the MC, and this is acceptable. For higher photon beam energy of 15 MV ($\text{field size}=10\times 10\;{\text{cm}}^{2}$) as shown in Figs. 4(a)–4(c), it is seen in Fig. 4(a) that the AAA appears to overestimate the dose slightly in the outer penumbra region (i.e., percentage dose between 50% and 20%) compared to the MC. The AAA appears to slightly overestimate the dose compared to the MC between 50% and 40%, and slightly underestimate between 40% and 20% when the depth of the profile is increased to 10 cm, as shown in Fig. 4(b). In Fig. 4(c), the AAA is found to slightly overestimate the dose at a depth of 15 cm compared to the MC. Positional deviations between the AAA/CCC and MC in the penumbra are within 2 mm in Fig. 4.

### C. Gamma dose distribution and dose‐volume histogram

Figures 5(a)–5(d) show gamma evaluation results by comparing the AAA and CCC to the MC. The 2D dose distributions (x‐z plane as seen in Fig. 1) using the 6 MV ($4\times 4\;{\text{cm}}^{2}$) and 15 MV ($7\times 7\;{\text{cm}}^{2}$) photon beams were used in the gamma dose comparisons. Figures 5(a) and 5(b) show the gamma dose distribution comparisons for the AAA and CCC with MC using the 6 MV photon beams, while Figs. 5(c) and 5(d) are corresponding comparisons for the 15 MV photon beams. The negative x‐axis represents the air region beyond the phantom lateral surface. This air space is needed in the gamma analysis because it allows the dose near the edge to search beyond for comparison. It should be noted that the isocenter of the photon beam was shifted 2 mm to the positive X direction pointed toward the phantom, as in Fig. 1. It is seen in Figs. 5(a) and 5(b) that deviations of the 2D dose distribution occur in both beam edges of the $4\times 4\;{\text{cm}}^{2}$ photon beams, and the deviation in the penumbra region is more significant than that of the air‐phantom surface. Since there is only about half photon beam in the phantom, the inadequate handling of the charge particle equilibrium for the convolution/superposition algorithm leads to a deviation of 2D dose distribution at the phantom lateral surface.^(^ ^38^ ^)^ Moreover, this shows that the dose deviations (AAA vs. MC and CCC vs. MC) at the phantom lateral surface are less significant than those at the penumbra region. For the 15 MV photon beams ($7\times 7\;{\text{cm}}^{2}$), the deviation of 2D dose distribution for the AAA at the penumbra region (Fig. 5(c) is seen to be more significant than that for the CCC (Fig. 5(d). However, the 2D dose deviations at the phantom lateral surfaces for both algorithms are found to be less significant when compared to the 2D dose deviations in the penumbra region.

Figures 6(a) and 6(b) show the DVHs of a skin slab (Fig. 1) with thickness of 2 mm for the 6 and 15 MV photon beams, respectively. The DVHs in Fig. 6 represent the dose uniformity within the skin volume, and the dose drop‐off region of the dose‐volume curve in the Figure reflects the dose distribution along the phantom lateral surface. The larger the width of the dose drop‐off region, the smaller is the dose gradient at the air‐phantom interface. In Fig. 6, both the AAA and CCC underestimate the width of the dose drop‐off region when compared to the MC. This means that both the AAA and CCC overestimate the dose uniformity in the skin, compared to the MC. Moreover, DVH slopes of the CCC in Fig. 6 are on the left of those of the AAA. This reflects a shaper dose drop‐off at the air‐phantom interface for the CCC, as shown in Figs. 3 and 4. In Fig. 6(a), DVHs of the CCC and MC agree better than the AAA. In Fig. 6(b), MC results are between those of the AAA and CCC, with the CCC mainly underestimating and AAA overestimating doses in the width of the dose drop‐off region.

### D. Phantom skin profile for nonoblique and oblique photon beams

Table I shows the percentage differences of the mean dose calculated for depth ranges of 5–20 cm (gantry angles equal to 0° and 5°) or 5–15 cm (gantry angle equal to 45°) for the phantom skin profiles. Dose differences in Table I are based on differences between doses calculated by the AAA/CCC and MC for the 6 and 15 MV photon beams with different field sizes and gantry angles.

**Table I:** Percentage differences of the mean dose and SDs calculated for depth ranges of 5–20 (gantry angles equal to 0° and 5°) or 5–15 cm (gantry angle equal to 45°) using the 6 and 15 MV photon beams with the calculated configuration as in Fig. 1. A negative value in the table means an underestimation of dose by the AAA and CCC compared to the MC. The field sizes are 4×4 (6 MV), 7×7 (15 MV), 10×10 and $20\times 20\;{\text{cm}}^{2}$ and gantry angles are 0°, 5° and 45°.

	4×4 or $7\times 7\;{\text{cm}}^{2}$						$10\times 10\;{cm}^{2}$						$20\times 20\;{cm}^{2}$					
*Energy*	*0°*		*5°*		*45°*		*0°*		*5°*		*45°*		*0°*		*5°*		*45°*	
	*AAA%*	*CCC%*	*AAA%*	*CCC%*	*AAA%*	*CCC%*	*AAA%*	*CCC%*	*AAA%*	*CCC%*	*AAA%*	*CCC%*	*AAA%*	*CCC%*	*AAA%*	*CCC%*	*AAA%*	*CCC%*
6 MV	10.5±1.3	3.4±0.9	−15.1±3.6	−3.7±1.5	6.4±0.6	21.3±0.5	7.6±2.6	2.1±1.3	−3.2±1.2	1.8±3.8	9.0±4.5	18.3±7.0	16.3±2.1	6.7±2.1	3.7±2.7	3.2±1.6	17.6±2.3	12.5±2.4
15 MV	7.8±1.6	4.6±1.7	−12.0±3.5	−7.6±2.0	4.0±0.7	9.1±0.9	5.5±1.2	1.7±1.4	0.4±2.2	0.6±0.4	13.5±2.8	19.6±2.1	18.0±1.3	8.3±1.8	4.0±1.7	−3.9±1.7	22.6±3.0	21.4±3.9

#### D.1 Nonoblique photon beams

For gantry angle equal to 0° in Table I, it is seen that both the AAA and CCC overestimate the phantom skin profiles for the 6 MV photon beams ($4\times 4\;{\text{cm}}^{2}$). The mean dose differences (%) are 10.5%±1.3% and 3.4%±0.9% for the AAA and CCC, when compared to the MC, respectively. It should be noted that the tolerance of complex beam geometry for TPS is 5%.^(^ ^39^ ^)^ When the field size of the beam is increased to $10\times 10\;{\text{cm}}^{2}$, the agreement between the AAA/CCC and MC becomes better, and the mean depth dose differences are 7.6%±2.6% and 2.1%±1.3% for the AAA and CCC as shown in Table I, respectively. This is because the normalization point of the $4\times 4\;{\text{cm}}^{2}$ field is very close to the beam edge. When the photon beam energy is increased to 15 MV, overestimations of the phantom skin profiles by the AAA and CCC occur as for the 6 MV. For the 15 MV photon beams, the mean dose differences for the AAA and CCC are 7.8% and 4.6% ($7\times 7\;{\text{cm}}^{2}$), 5.5% and 1.7% ($10\times 10\;{\text{cm}}^{2}$), and 18% and 8.3% ($20\times 20\;{\text{cm}}^{2}$), respectively. The dose differences of the 15 MV photon beam are worse than those of the 6 MV. This is due to the longer electron paths produced by the higher beam energy of 15 MV. Since the electron side‐scatter is related to its path length, the overestimation of the phantom skin profile depends on the photon beam energy.

#### D.2 Oblique photon beams

For oblique photon beams at 5°, it is seen in Table I for the 6 MV ($4\times 4\;{\text{cm}}^{2}$) that both the AAA and CCC underestimate the phantom skin profile compared to the MC. The mean depth dose differences are −15.1%±3.6% and −3.7%±1.5% for the AAA and CCC with beam angle equal to 5°, respectively. When the field size is increased to $10\times 10\;{\text{cm}}^{2}$, the mean depth dose differences are −3.2%±1.2% for the AAA and 1.8%±3.8% for the CCC. Similar dosimetric characteristics for the phantom skin profiles can be found for the 15 MV photon beams, with mean dose differences for the small field ($7\times 7\;{\text{cm}}^{2}$) equal to −12%±3.5% and −7.6%±2% for the AAA and CCC with beam angle equal to 5°, respectively. It can be seen in Table I that both the 6 and 15 MV photon beams have smaller deviations in the mean dose differences for the $10\times 10\;{\text{cm}}^{2}$ than for smaller or larger fields. The underestimation of dose for small fields predicted by the AAA and CCC may be due to the fact that when the beam is tilted slightly anticlockwise, the beam edge in the phantom is moved away from the normalization point. This results in more electron scatter within the beam in relation to the normalization point and, hence, a lower relative dose compared to that with the same field size and gantry angle equal to 0°. Moreover, when the gantry is tilted 5°, the beam arrangement is very close to tangential. Therefore, contiguous pencil beams or energy fluences would enter the phantom lateral surface with very different SSDs and the discretization effects mentioned above become evident. This also affects the determination of the TERMA through the ray‐tracing technique to incorporate the effects of the air‐phantom interface on lateral scatter.

For oblique photon beams at 45°, the CAX crosses the phantom skin profile. It can be seen that the discretization effect is more serious in a larger oblique incidence of 45° than 5° showing higher depth dose differences with different field sizes, as shown in Table I. For the $20\times 20\;{\text{cm}}^{2}$ field, the mean dose differences of the AAA and CCC are 17.6%±2.3% and 12.5%±2.4% for the 6 MV photon beams, and 22.6%±3.0% and 21.4%±3.9% for the 18MV. In addition to the discretization effect, the overestimations of doses from a lower (air) to higher (water) density medium can also be found in lung‐water interface with the lung relative electron density <0.1.^(^ ^20^ ^)^ This is due to the overestimation of electron backscatter from the higher density medium in the convolution/superposition algorithm.

## IV. CONCLUSIONS

Dose calculations of the AAA and CCC under different tangential beam geometries were evaluated in a solid water phantom using the MC simulation. Photon beams of 6 and 15 MV with field sizes of 4×4 (6 MV), 7×7 (15 MV), 10×10 and $20\times 20\;{\text{cm}}^{2}$ were used. Apart from photon beam angle of 0°, beams were turned 5° and 45° around the isocenter located at a distance of 2 mm from the phantom lateral surface. The horizontal dose profiles with different depths, phantom skin profiles parallel to the CAX, results of gamma dose distribution comparisons and DVHs of skin slab were determined. It is found that there are dosimetric deviations of the AAA and CCC results when compared to the MC in a tangential‐like geometry. Moreover, both AAA and CCC cannot predict doses reliably at depth less than 2 mm. Future work includes studying the dosimetric dependence of the skin profile on the tangential beam geometry using small segmental photon fields for IMRT. For radiation treatments in the chest wall, breast and sarcoma, their effectiveness may be compromised by replying only on the TPS data. This dosimetric issue should be a concern when an accurate skin dose prediction is needed, in particular when the tumor is near or at the patient's surface.

## ACKNOWLEDGEMENTS

The authors would like to thank Dr Rob Barnett for his support on studies of dose calculations using the Eclipse and ${\text{Pinnacle}}^{3}$ TPS in the Grand River Hospital. The gamma tool provided by Drs. D. A. Low and J. F. Dempsey for the gamma dose distribution analysis is kindly acknowledged. We would also like to thank all physics staff contributing to the commissioning of the TPSs in the Grand River Hospital.

## Supporting information

Supplementary Material

Supplementary Material
