# Supplementary figures and images for: Dosimetry of oblique tangential photon beams calculated by superposition/convolution algorithms: a Monte Carlo evaluation
Source: J Appl Clin Med Phys. 2010 Nov 3;12(1):108–21. doi: 10.1120/jacmp.v12i1.3424 (PMC5718594; doi:10.1120/jacmp.v12i1.3424)

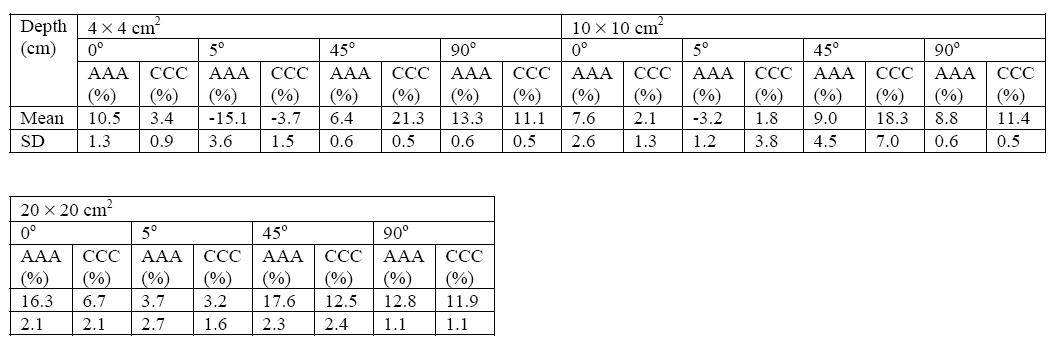

Supplement: Supplementary file 1 — Supplementary Material [file ACM2-12-108-s001.jpg]

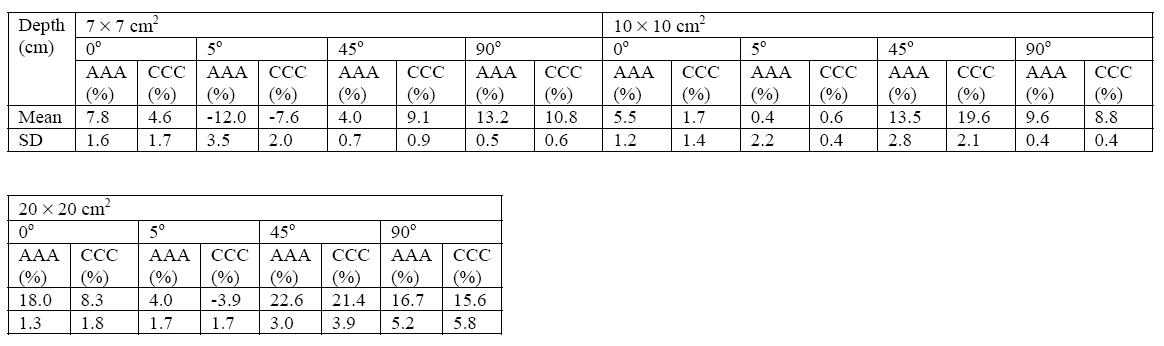

Supplement: Supplementary file 2 — Supplementary Material [file ACM2-12-108-s002.jpg]
